# Patient preferences for antihypertensive medication dispensing trade-offs in cardiovascular disease prevention: national survey in Australia

**DOI:** 10.1136/bmjopen-2026-122691

**Published:** 2026-10-12

**Authors:** Carissa Bonner, Michael Anthony Fajardo, Rachael Keast, Prerna Ganguli, Emily Atkins, Niamh Chapman, Kristie Weir, Anthony Rodgers, Aletta E Schutte

**Affiliations:** 1 Leeder Centre for Health Policy, University of Sydney, Sydney, New South Wales, Australia; 2 Sydney Health Literacy Lab, University of Sydney, Sydney, New South Wales, Australia; 3 The George Institute for Global Health, Sydney, New South Wales, Australia; 4 Faculty of Medicine and Health, School of Health Sciences, University of Sydney, Sydney, New South Wales, Australia; 5 School of Population Health, University of New South Wales—Kensington Campus, Sydney, New South Wales, Australia

**Keywords:** Blood Pressure, Hypertension, Prescriptions, Health economics

## Abstract

**Abstract:**

**Objectives:**

This study investigated predictors of consumer preferences for different antihypertensive dispensing policies in Australia.

**Design:**

Cross-sectional survey.

**Setting:**

An online national survey in Australia between 27 November 2024 and 20 December 2024.

**Participants:**

2054 adults taking antihypertensives completed the online survey experiment. Quota sampling was used to ensure sufficient diversity in age, gender and education groups.

**Primary and secondary outcome measures:**

The primary outcome was participant preference between dispensing options. The survey included six forced choices between dispensing policy options that varied on (1) delivery method, (2) dispensing duration and (3) cost. For each choice set, participants chose 1 out of 2 dispensing options that either indicated a preference for pharmacy pickup over mail or longer over shorter dispensing duration. Analyses involved regression models for each forced-choice question.

**Results:**

Overall, participants preferred delivery through pharmacies rather than post (range 75%–82% across two choice sets). When shown cheaper annual costs, they preferred longer dispensing of antihypertensives (range 69%–92% across four choice sets). Preference for shorter dispensing despite higher annual costs was predicted by access to government support for high medication costs. For non-concession card holders, this was a significant predictor (p<0.004) across 3/4 choice sets (OR range 1.3–4.1). For concession card holders, this was a significant predictor (p<0.002) across all four choice sets (OR range 2.3–4.3). Living in a major city, having a university education, not being female, being under 65 years of age and having a higher income predicted shorter dispensing preference in some but not all choice sets.

**Conclusions:**

Government support for medication costs is a significant consideration when changing dispensing policy in Australia, which may differentially affect concession card holders. Implementing changes to antihypertensive dispensing requires clear communication about cost implications, including the impact on reaching government thresholds for financial assistance.

**STRENGTHS AND LIMITATIONS OF THIS STUDY**

- This study presents new evidence on consumer dispensing preferences from a large national sample of 2054 people in Australia who take antihypertensives, enabling us to explore multiple factors that predict dispensing preference.
- The study design and forced choice presentation were informed by a diverse group of consumer advisors, and findings are relevant to policymakers.
- The study design was a self-reported preference survey, providing an overall indication of preference and price sensitivity, but no inferences can be drawn about trade-offs between costs and method of delivery.
- Stated preferences may not reflect real-world behaviour.
- The financial context in Australia may not generalise to other countries.

## Introduction

 Uncontrolled hypertension is the leading cause of preventable cardiovascular disease experienced by more than 1.4 billion adults globally, with less than half receiving recommended medication.^1^ Only four countries achieved greater than 50% control rates of hypertension in 2024.^2^ The WHO has identified access to antihypertensives as the cornerstone of hypertension control.^3^

A key component of access to antihypertensives is dispensing duration. Dispensing duration refers to the length of time that a medication can be obtained from a pharmacy with a single script usually prescribed by a general practitioner; in other words, the amount of medication that can be dispensed at one time, such as a 30-day supply or a 90-day supply. In Australia, costs for dispensing antihypertensives account for approximately one-third of all hypertension treatment costs, including out-of-pocket and health system costs.^4^ Dispensing duration for many chronic medications, including antihypertensives, was extended in 2023 in Australia from 30 to 60 days to save costs for the government and patients.^5^ However, uptake of longer dispensing has been slow.^6^ International dispensing durations vary from 30 to 180 days,^7^ with the recent 2025 Global Report on Hypertension from the WHO recommending 90-day dispensing as the optimal point for increased medication adherence.^3^

Longer dispensing durations may improve adherence by reducing the burden on the patient to refill their medications frequently, and reduced dispensing fees may be associated with lower costs to patients and the healthcare system.^7^ A large retrospective US claims analysis found 90-day scripts were associated with a 27% improvement in adherence.^8 9^ In Thailand, changing from a 30-day to 90-day dispensing duration improved adherence to statins and diabetes medication.^10^ Potential risks of longer dispensing durations may include medicine wastage^9^ and reduced health professional contact to guide management.^7^ Research on this issue is limited for the Australian context, where dispensing changes may also affect medication subsidies from the government.^11^ There is a lack of evidence on patient preferences or factors that influence patient decision-making regarding dispensing. This online, national survey explored patient preferences for antihypertensive dispensing trade-offs between delivery method, dispensing duration and cost, to inform a trial of alternative dispensing policies.

## Methods

### Study design

This was an online self-reported preference survey of a large national sample of people in Australia taking antihypertensives. The survey was completed using Qualtrics with recruitment through Dynata—a large market research company with more than 600 000 online Australian panel members aged older than 18 years. Respondents were eligible to participate in the study if they indicated they were at least 18 years old and taking antihypertensives. Quota sampling was used to ensure sufficient diversity in age, gender and education groups during the recruitment period between 27 November 2024 and 20 December 2024, before relaxing quotas to achieve the target sample of 2000. We aimed to detect preference differences between concession card holders and non-concession card holders, assuming a difference of 5% would equate to 435 respondents per group (total 870).

### Choice sets

The primary focus of this paper is predictors of the preferences within each of 6 dispensing choice sets (table 1, figure 1). General preferences are reported in brief research letter format elsewhere, to inform local policy makers.^12^ Choice sets 1 and 2 highlight differences in preferences between pharmacy pickup and postal delivery options (with equivalent dispensing duration/cost options). Choice sets 3–6 highlight dispensing duration preferences (keeping pharmacy pickup or postal delivery options equivalent, while varying duration). Choice sets 3 and 6 compared 60 versus 90 pills dispensing options, whereas choice sets 4 and 5 compared 30 versus 120 pills dispensing options for pharmacy and delivery, respectively. Concession card holders had consistent costs per order irrespective of size, and non-concession card holders had higher costs for larger dispensing options. For both concession and non-concession card holders, total annual costs were always cheaper for larger dispensing options. Further detail is available in the online supplemental materials.

**Table 1:** Description of each forced choice set

Choice set	Attributes	Concession		No concession	
		Option A	Option B	Option A	Option B
Choice 1 Pharmacy vs postal for 60-day supply	Delivery No of pills $A per order $A per year	**Pharmacy** 60 $A7.70 $A46.20	**Postal** 60 $A7.70 $A46.20	**Pharmacy** 60 $A18.40 $A110.40	**Postal** 60 $A18.40 $A110.40
Choice 2 Pharmacy vs postal for 90-day supply	Delivery No of pills $A per order $A per year	**Pharmacy** 90 $A7.70 $A30.80	**Postal** 90 $A7.70 $A30.80	**Pharmacy** 90 $A20.90 $A83.60	**Postal** 90 $A20.90 $A83.60
Choice 3 60- vs 90-day supply via pharmacy	Delivery No of pills $A per order $A per year	Pharmacy **60** $A7.70 **$A46.20**	Pharmacy **90** $A7.70 **$A30.80**	Pharmacy **60** **$A18.40** **$A110.40**	Pharmacy **90** **$A20.90** **$A83.60**
Choice 4 30- vs 120-day supply by post	Delivery No of pills $A per order $A per year	Postal **30** $A7.70 **$A92.40**	Postal **120** $A7.70 **$A23.10**	Postal **30** **$A15.90** **$A190.80**	Postal **120** **$A23.40** **$A70.20**
Choice 5 30- vs 120-day supply via pharmacy	Delivery No of pills $A per order $A per year	Pharmacy **30** $A7.70 **$A92.40**	Pharmacy **120** $A7.70 **$A23.10**	Pharmacy **30** **$A15.90** **$A190.80**	Pharmacy **120** **$A23.40** **$A70.20**
Choice 6 60- vs 90-day supply by post	Delivery No of pills $A per order $A per year	Postal **60** $A7.70 **$A46.20**	Postal **90** $A7.70 **$A30.80**	Postal **60** **$A18.40** **$A110.40**	Postal **90** **$A20.90** **$A83.60**

Differences indicated in bold.

**Figure 1:**
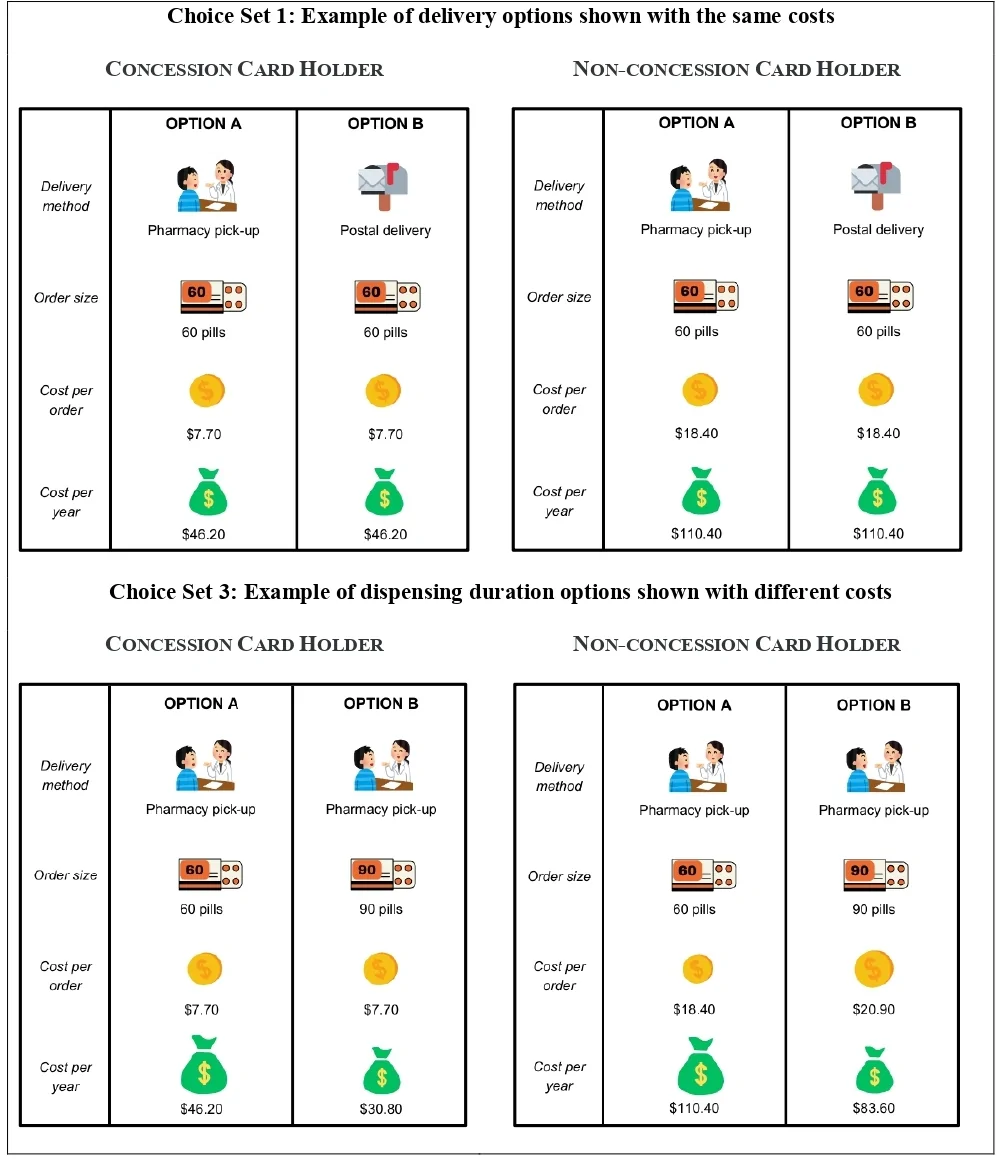
Example of choice sets for non-concession card holders and concession card holders.

### Patient and public involvement

The survey was codesigned with a consumer advisory group through the Sydney Health Literacy Lab, to elicit preferences for medication dispensing methods and intervals. This involved iterative discussion, development, testing and refinement with a diverse group of seven consumers (men and women aged between 30 and 85, with varying cardiovascular risk and medication experience, including coauthor PG).

### Survey information

The full survey and process are detailed in the online supplemental materials, and a short-format summary of the overall dispensing preferences is published elsewhere.^12^ Consent was provided by answering a survey question before proceeding. The focus of this paper is the predictors of the six forced-choice responses, where alternative dispensing options were presented with estimated costs, which were different for concession and non-concession card holders. Patient characteristic questions included: age, gender, education, regionality, concession card holder status, whether respondents reached Pharmaceutical Benefits Scheme (PBS) Safety Net in a calendar year (see Australian healthcare context section below), amount of different medications (prescribed and not prescribed) taken per week, whether their current medication collection was typically via post or picked up at the pharmacy, self-reported blood pressure information, and perceived affordability of medicine. If a respondent indicated ‘Don’t Know’ to concession card holder status, they were considered to be a non-concession card holder for this part of the survey. The separation was important because in Australia concession card holders can purchase medications at a lower price. Costs used were presented based on PBS prices in May 2024. Respondents viewed one forced-choice set at a time.

### Australian healthcare context

In Australia, the PBS Safety Net provides the yearly spending threshold, up to which patients pay the full cost of medications within a calendar year.^11^ In 2024, when this study was designed, the threshold was $A277.20 for concession card holders and $A1647.90 for non-concession card holders and is annually updated to align with the Consumer Price Index on 1 January. In May 2026, the threshold remains the same for concession card holders but has increased to $A1748.20 for general patients. Once this threshold is reached, patients can apply for a PBS Safety Net card to get access to cheaper ($A7.70 copay) or free medicines for the rest of the calendar year. Cheaper medicine is also accessible through Concession Cards, which are eligible for Australian pensioners and low-income individuals.^13^

### Statistical analysis

Descriptive statistics for sample characteristics were calculated using frequencies and means. Logistic regressions were conducted for each choice set to assess predictors of preferences for options in each choice set. This included the following categorical binary predictors: whether the PBS safety threshold was reached, whether participants lived in a major city (vs not), preference for 1–2 months dispensing (vs 3–6 months), preference for pharmacy pickup options (vs delivery), university education (vs no university education), female (vs male), under 65-year-olds (vs 65 years and over) and weekly income as a continuous variable. Full model statistics and summary tables are provided in the online supplemental materials. All statistical analyses were conducted in SPSS V.29.^14^ For ease of interpretation, weekly income was treated as a continuous variable in logistic regression models despite it being collected as ordinal data. The linearity assumption for weekly income and age (in years) was assessed via a Box-Tidwell test and indicated that weekly income did not violate the linearity assumption, whereas age (in years) did in some models.

Checks of model fit between age in years and age groups (18–44, 45–65 and 65+) showed marginal changes in model fit statistics (−2LL, pseudo R^2^) across models despite violating the linearity assumption; thus, for ease of interpretation, age was treated as a binary categorical variable in all models. The cut point for age was guided by policy decisions, as 65 years is an important change in access to medications and screening decisions.

Similarly, education was dichotomised to university education versus no university education and regional/remoteness area was dichotomised to lives in a major city versus does not live in a major city.

Postcode was used to estimate regional/remoteness area using the ABS Data 2017 to 2016 Remoteness Area File.^15^ Where there were multiple entries for postcodes in the ABS dataset, the postcode with the largest portion was used to indicate remoteness area. Case-wise exclusion of data was conducted within analyses. Final sample sizes per analysis are indicated.

## Results

### Participants

5545 respondents from Dynata’s panel consented to the study. After respondents completed quota and eligibility questions, a final sample of 2054 respondents completed the online survey experiment. Table 2 provides the sample characteristics. While broadly consistent with the Australian population, the age range is higher for those taking antihypertensives. There were some discrepancies compared with Australian census data, where the proportion living in a major city is 67% in the general population,^16^ compared with 77% in our sample. For income, Australian census data shows that 60% of the population has a weekly income less than $A1000,^17^ compared with 40% in our sample. However, due to the large sample size, we were still able to explore the influence of these factors in analyses.

**Table 2:** Characteristics of the survey sample (n=2054)

Age (category)	Count	%
18–44 years	449	21.9
45–64 years	693	33.7
65 years or more	912	44.4
Gender identity (missing=4)	Count	%
Male	1033	50.4
Female	1014	49.5
Trans and/or gender diverse	0	0.0
I use a different term (please specify)	1	0.0
I’d prefer not to say	2	0.1
Education (missing=4)	Count	%
Below year 10 including certificate I/II	110	5.4
Year 10 or equivalent	160	7.8
Year 11–12	422	12.8
Certificate III/IV	333	16.2
Diploma/associate degree	241	11.8
University degree (eg, bachelor’s)	619	30.2
Graduate diploma or graduate certificate	144	7.0
Postgraduate (eg, master’s)	181	8.8
Weekly income (missing=13)	Count	%
No income	40	2.0
$A1–$A499	183	9
$A500–$A649	212	10.4
$A650–$A799	153	7.5
$A800–$A999	245	12.0
$A1000–$A1249	288	14.1
$A1250–$A1499	217	10.6
$A1500–$A1749	136	6.7
$A1750–$A1999	113	5.5
$A2000–$A2999	217	10.6
$A3000+	237	11.6
Concession card (missing=1)	Count	%
Yes	1335	65.0
No	711	34.6
Don’t know	7	0.3
Reached PBS safety net in a calendar year (missing=4)	Count	%
Yes	1044	50.9
No	810	39.5
Don’t know	196	9.6
Remoteness area (19 missing or invalid postcodes)	Count	%
Major city	1576	77.3
Inner regional	313	15.4
Outer regional	135	6.6
Remote	6	0.3
Very remote	5	0.2

PBS, Pharmaceutical Benefits Scheme.

### Preferred options

Most respondents indicated an overall preference to obtain antihypertensives directly from the pharmacy as indicated in choice sets 1 and 2; and preferred longer dispensing durations when cheaper annual costs were shown, as indicated in choice sets 3–6 (figure 2).

**Figure 2:**
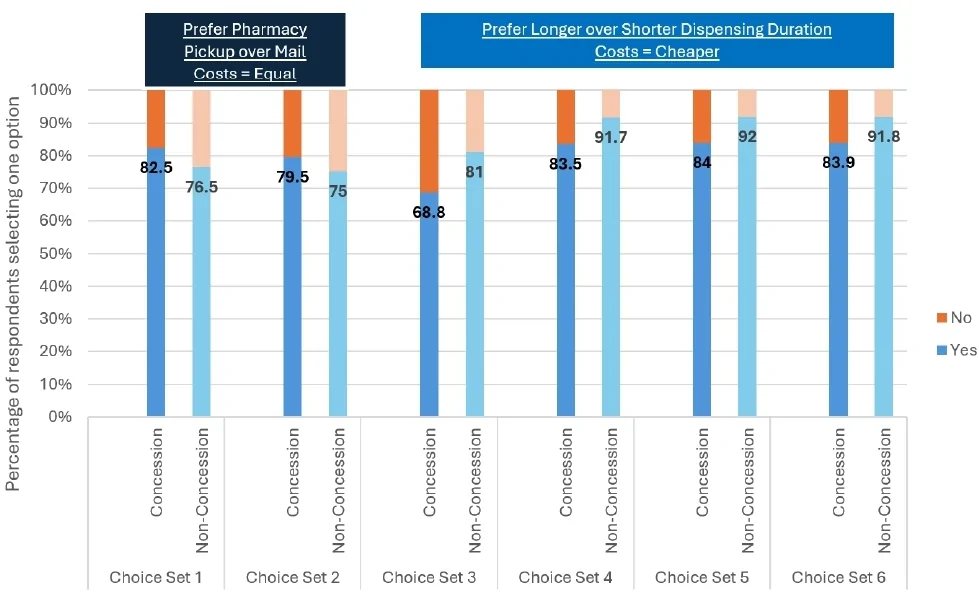
Percentage of respondents preferring pharmacy pickup (choice sets 1–2) and longer dispensing (choice sets 3–6) for concession versus non-concession card holders. Choice sets 1 and 2 indicate the percentage of respondents choosing pharmacy pickup over mail delivery when costs are equal; choice sets 3–6 indicate the percentage of respondents choosing longer dispensing duration over shorter dispensing when annual costs are cheaper.

### Pharmacy versus postal delivery

Factors that predicted preferences between pharmacy pickup and mail delivery were similar between concession and non-concession card holders (see table 3). Prior preference for the current policy of shorter dispensing with pharmacy pickup was a significant predictor of selecting pharmacy pickup options, irrespective of 60- or 90-day dispensing. Younger respondents aged under 65 years were more likely to prefer postal delivery options, but this preference did not reach statistical significance for non-concession card holders in choice set 1. There was also a clear difference between concession and non-concession card holders, where non-concession card holders with higher income preferred postal delivery; whereas income was not a significant predictor of postal delivery versus pharmacy pickup preference for concession card holders. The area under the curve for the models suggests each model predicted selected options at a level higher than chance.

**Table 3:** Predictors of preference for pharmacy pickup over postal delivery (choice sets 1–2)

Binary predictors		Choice sets with pharmacy pickup preference as the outcome of interest			
		1: both 60-day dispensing		2: both 90-day dispensing	
		Concession n*=*1187	Non-concession n=599	Concession n*=*1188	Non-concession n=599
Reached PBS safety net threshold	OR	1.10	1.40	1.12	1.09
	95% CI	0.70 to 1.73	0.80 to 2.44	0.74 to 1.68	0.64 to 1.84
	P	0.675	0.242	0.589	0.751
Lives in a major city vs regional area	OR	1.03	1.02	0.96	0.75
	95% CI	0.65 to 1.62	0.57 to 1.84	0.63 to 1.47	0.41 to 1.37
	P	0.915	0.941	0.864	0.346
Prefers shorter (30–60 day) dispensing vs longer duration	OR	**2.36**	**2.34**	**2.14**	**1.90**
	95% CI	**1.62 to 3.42**	**1.47 to 3.41**	**1.52 to 3.01**	**1.26 to 2.87**
	P	**<0.001**	**<0.001**	**<0.001**	**0.002**
Prefers pharmacy pickup vs postal delivery	OR	**19.94**	**16.06**	**13.65**	**14.50**
	95% CI	**12.73 to 31.22**	**7.65 to 33.70**	**8.90 to 20.93**	**6.93 to 30.35**
	P	**<0.001**	**<0.001**	**<0.001**	**<0.001**
University-educated vs school only	OR	1.03	1.01	0.79	0.96
	95% CI	0.67 to 1.59	0.65 to 1.58	0.53 to 1.16	0.62 to 1.48
	P	0.882	0.958	0.224	0.857
Females vs males and other genders	OR	0.95	0.75	0.98	0.71
	95% CI	0.65 to 1.38	0.49 to 1.15	0.70 to 1.38	0.47 to 1.07
	P	0.795	0.185	0.906	0.101
Under 65 years of age vs 65+ years	OR	**0.64**	0.61	**0.41**	**0.55**
	95% CI	**0.44 to 0.95**	0.35 to 1.05	**0.29 to 0.59**	**0.32 to 0.94**
	P	**0.025**	0.073	**<0.001**	**0.029**
Weekly income (continuous)	OR	1.01	**0.88**	1.05	**0.90**
	95% CI	0.95 to 1.08	**0.81 to 0.96**	0.93 to 1.11	**0.3 to 0.98**
	P	0.707	**0.003**	0.154	**0.012**
	AUC	0.816	0.737	0.817	0.732

Bolded cells indicate significant predictors at p<0.05, with ORs >1 indicating preference for pharmacy pickup and ORs <1 indicating preference for postal delivery.

PBS, Pharmaceutical Benefits Scheme.

### Shorter versus longer dispensing

Most respondents preferred longer dispensing with cheaper annual costs as per the overall frequencies (see figure 2). Factors that predicted the alternative preference for *shorter* dispensing, with higher annual costs but cheaper cost per order, were similar for concession and non-concession card holders (see table 4). Participants who had high enough medication costs to reach the PBS safety net threshold, did not identify as female or were under 65 years of age were more likely to prefer the shorter dispensing option. For concession card holders, those who lived in a major city, had a university education and had a higher income were also more likely to prefer the shorter dispensing option. Choice set 3, which compares 60-day to 90-day dispensing in pharmacies, appears to be an anomaly as it does not follow these trends. The area under the curve for the models suggests each model predicts selected options at a level higher than chance, except for choice set 3 (60- vs 90-day dispensing with pharmacy pickup).

**Table 4:** Predictors of preference for shorter dispensing duration despite higher annual cost (choice sets 3–6)

Binary predictor level of interest		Choice sets with preference for shorter dispensing duration (despite higher annual cost) as outcome of interest							
		3: 60 vs 90 day; both pharmacy pickup		4: 30 vs 120 day; both postal delivery		5: 30 vs 120 day; both pharmacy pickup		6: 60 vs 90 day; both postal delivery	
		Concession n*=*1188	Non-concession n=599	Concession n*=*1189	Non-concession n=598	Concession n*=*1186	Non-concession n=597	Concession n*=*1188	Non-concession n=597
Reached PBS safety net threshold	OR	**2.64**	1.35	**2.28**	**4.14**	**2.29**	**3.41**	**4.30**	**2.59**
	95% CI	**1.88 to 3.69**	0.82 to 2.22	**1.39 to 3.74**	**2.28 to 7.53**	**1.39 to 3.78**	**1.85 to 6.29**	**2.31 to 8.02**	**1.37 to 4.89**
	P	**<0.001**	0.246	**0.001**	**<0.001**	**0.001**	**<0.001**	**<0.001**	**0.003**
Lives in a major city vs regional area	OR	1.18	1.27	**2.03**	1.27	**1.91**	1.37	**2.15**	1.87
	95% CI	0.87–1.60	0.71–2.25	**1.24–3.32**	0.55–2.91	**1.17–3.11**	0.58–3.27	**1.28–3.61**	0.70–5.01
	P	0.282	0.421	**0.005**	0.575	**0.009**	0.475	**0.004**	0.212
Prefers shorter (30–60 day) dispensing vs longer duration	OR	0.99	**1.84**	0.72	1.59	0.73	1.62	0.79	1.80
	95% CI	0.75–1.61	**1.18–2.88**	0.51–1.01	0.86–2.96	0.52–1.03	0.86–3.05	0.55–1.13	0.94–3.42
	P	0.944	**0.007**	0.061	0.141	0.076	0.135	0.193	0.075
Prefers pharmacy pickup vs postal delivery	OR	1.11	2.14	**0.61**	1.12	0.72	1.35	**0.54**	1.42
	95% CI	0.76–1.62	0.86–5.28	**0.41–0.92**	0.43–2.94	0.47–1.09	0.49–3.76	**0.35–0.81**	0.51–3.93
	P	0.608	0.101	**0.019**	0.815	0.122	0.563	**0.003**	0.502
University educated vs school only	OR	**1.50**	0.91	**1.93**	1.01	**2.13**	1.41	**1.99**	1.66
	95% CI	**1.12–1.99**	0.59–1.40	**1.31–2.83**	0.54–1.90	**1.44–3.14**	0.74–2.70	**1.33–2.96**	0.85–3.22
	P	**0.006**	0.673	**<0.001**	0.976	**<0.001**	0.295	**<0.001**	0.135
Females vs male/other genders	OR	0.78	1.23	0.93	0.81	**0.55**	0.58	**0.64**	**0.60**
	95% CI	0.84–1.44	0.81–1.85	0.66–1.30	0.45–1.46	**0.39–0.79**	0.32–1.07	**0.45–0.92**	**0.33–1.10**
	P	0.484	0.337	0.660	0.488	**<0.001**	0.081	**0.014**	**0.002**
Under 65 years of age vs 65+ years	OR	1.10	1.53	**2.08**	**3.74**	**2.63**	**3.11**	**2.52**	**6.80**
	95% CI	0.84–1.44	0.92–2.54	**1.46–2.97**	**1.43–9.80**	**1.82–3.78**	**1.27–7.61**	**1.74–3.65**	**2.05–22.53**
	P	0.484	0.100	**<0.001**	**0.007**	**<0.001**	**0.013**	**<0.001**	**0.002**
Weekly income (continuous)	OR	0.99	0.96	**1.09**	0.96	**1.07**	0.94	**1.12**	0.94
	95% CI	0.95–1.04	0.90–1.03	**1.02–1.15**	0.87–1.06	**1.01–1.14**	0.85–1.04	**1.05–1.19**	0.85–1.04
	P	0.782	0.238	**0.009**	0.441	**0.031**	0.222	**<0.001**	0.221
	AUC	0.639	0.623	0.754	0.709	0.771	0.716	0.805	0.744

Bolded cells indicate significant predictors at p<0.05 with ORs >1 indicating preference shorter dispensing and greater annual cost and ORs <1 indicating preference for longer dispensing and lower annual cost.

## Discussion

This national survey across Australia shows that consumer preferences for antihypertensive dispensing are strongly influenced by cost considerations. Irrespective of dispensing duration and associated costs, at least 75% of respondents preferred pharmacy pickup across the two delivery-focused choice sets, when costs were equal. However, the majority of respondents preferred longer dispensing duration when cheaper annual cost was clearly displayed, across the choice sets. This suggests an overall preference to extend the current policy of 1–2 months dispensing duration, if annual consumer costs can be reduced.

However, specific groups may have different delivery and dispensing preferences. Younger and higher income participants were more likely to prefer postal delivery as an option regardless of price, suggesting some groups may benefit from easier access to regular medication supply. Respondents who generally reach the PBS safety net threshold were more likely to prefer shorter dispensing despite higher annual cost, irrespective of concession card holder status. This may reflect more of a focus on short-term out-of-pocket costs per order or less concern about annual costs due to reaching the government assistance threshold. Concession card holders who live in a major city or were university educated, and those aged under 65, were also more likely to prefer the status quo of shorter dispensing. In contrast, concession card holders with lower income, less education and regional locations appeared to be more influenced by the total annual cost, with a preference for longer dispensing.

These patterns are consistent with prior literature showing that medication preferences are shaped by a combination of cost sensitivity, accessibility, age and individual capacity. Consistent with prior work, financial considerations appear to shape decision-making among lower-income groups, who may prioritise options that minimise cumulative costs over time.^18^ In contrast, individuals with greater financial flexibility may place more weight on convenience and service flexibility, with evidence suggesting that younger and higher-educated populations are more adaptive to alternative models of care.^19–21^ Accessibility further moderates these preferences, as travel burden, geographical location and physical ability influence how feasible different medication access options are, particularly for those managing chronic conditions.^7 22^ Importantly, while some groups preferred shorter dispensing, existing evidence indicates that longer dispensing intervals are associated with improved adherence for antihypertensive and other chronic medications.^9 23 24^ This highlights a potential divergence between user preference and adherence-optimising practices, suggesting that policy decisions should account for both behavioural preferences and clinical effectiveness when designing medication access models.

### Strengths and limitations

This study represents a large sample of people in Australia who take antihypertensives, enabling us to explore multiple factors that predict dispensing preference. The study design and forced-choice presentation were informed by a diverse group of consumer advisors. However, the cross-sectional nature of this study cannot confidently remove the effect of unknown factors that influence these decisions. The study provides an overall indication of preference and price sensitivity, but no inferences can be drawn about trade-offs between costs and method of delivery. As an exploratory study, we conducted multiple analyses without correction. Using a Bonferroni correction for six analyses within concession card versus non-concession card holders’ groups identifies 0.008 as the level for significance, which only marginally changes significance interpretation within a couple of sets. This suggests that overall patterns are unlikely to be due to type 1 error. Although our sample included substantial demographic diversity, we acknowledge that it is not fully representative, given that all participants were willing and able to participate in an online survey. By limiting the sample to online participants, we potentially excluded participants with low computer literacy and digital health literacy. Finally, the decisions individuals make in a vignette may not fully reflect their real-life choices, and the financial context in Australia may not generalise to other countries.

### Future directions

This study provides insights for future policymakers and researchers in exploring demographic factors in Australia to consider when implementing changes to medication dispensing duration. The results have informed the design of a large national trial to test the effect of longer dispensing duration in Australia,^24^ which will include measures of medication adherence and blood pressure control. Preferences may vary in terms of regionality, age and financial factors, requiring careful consideration and communication of policy changes. Additional research exploring the reasons for differences between demographic groups and concession versus non-concession card holders would further support policymakers. In discussions with the consumer advisory panel, members noted that the results were different from what was expected while designing the survey, in that many consumers have a preference towards longer dispensing durations for antihypertensives instead of the status quo when costs are reduced. They also suggested that since the results show a strong preference towards cheaper annual dispensing costs overall, it would be helpful to consider an economic analysis for the national trial. The consumer advisors will be involved in coding open responses about dispensing preferences for a future qualitative paper on the reasons for different preferences. This study focuses on opportunity barriers to adherence in terms of costs and ease of access. Additional interventions would be needed to address motivational barriers to adherence, such as experience of side effects or low perceived risk.

### Conclusion

The consumer preferences in this study suggest that reducing antihypertensive costs through increased dispensing duration is a reasonable policy change to consider in Australia. As with any change, there needs to be due consideration of individual preferences, and how such a change may differentially affect groups that receive government subsidies for medication. The initial preference for the status quo of frequent pharmacy pickup every 1–2 months changed when offset by cost savings through longer dispensing. This requires careful consideration of financial implications for government support schemes. Consumer preferences need to be considered in modelling the potential uptake of dispensing policy changes in future.

## Supplementary material

10.1136/bmjopen-2026-122691 online supplemental file 1

## Data Availability

Data are available upon reasonable request.

## References

[1] Zhou B, Carrillo-Larco RM, Danaei G (2021). Worldwide trends in hypertension prevalence and progress in treatment and control from 1990 to 2019: a pooled analysis of 1201 population-representative studies with 104 million participants. The Lancet.

[2] World Health Organization (2025). Global report on hypertension 2025: high stakes - turning evidence into action.

[3] World Health Organization (2023). Global report on hypertension: the race against a silent killer.

[4] Atkins ER, Nguyen LH, Chatterton ML (2024). The cost of treating hypertension in Australia, 2012-22: an economic analysis. Med J Aust.

[5] Australian Government Department of Health, D.a.A Learn more about PBS medicines and 60-day prescriptions. https://www.health.gov.au/cheaper-medicines.

[6] Wang T, Nguyen HL, Tan I (2026). Uptake and Costs of the 60-Day Dispensing Policy for Antihypertensive Medicines in Australia: A Mixed Methods Study. Med J Aust.

[7] af Geijerstam P, Falster MO, Chalmers J (2024). Prescription and dispensing duration of medicines for hypertension and other chronic conditions: a review of international policies and evidence to inform the Australian setting. Hypertens Res.

[8] Hermes M, Gleason P, Starner C (2010). Adherence to chronic medication therapy associated with 90-day supplies compared with 30-day supplies. J Manag Care Pharm.

[9] King S, Miani C, Exley J (2018). Impact of issuing longer- versus shorter-duration prescriptions: a systematic review. Br J Gen Pract.

[10] Jarujumrus I, Taychakhoonavudh S (2023). Impact of prescription length supply policy on patient medication adherence in Thailand. BMC Health Serv Res.

[11] Services Australia (2026). PBS safety net thresholds. https://www.servicesaustralia.gov.au/pbs-safety-net-thresholds?amp%3B_amp%3Butm_source=chatgpt.com&context=22016.

[12] Bonner C, Fajardo MA, Keast RM (2025). Medication delivery and dispensing interval preferences of people who use antihypertensive medications in Australia: a survey study. Med J Aust.

[13] Services Australia (2025). Concession and health care cards. https://www.servicesaustralia.gov.au/concession-and-health-care-cards?context=60091&utm_source=chatgpt.com.

[14] IBM Corp (2022). IBM SPSS *statistics for windows (v*ersion 29.0).

[15] Australian Bureau of Statistics (2016). 2017 postcode to 2016 remoteness area.

[16] Australian Bureau of Statistics (2021). Location: census.

[17] Australian Bureau of Statistics (2021). Income and work: census.

[18] McMillan SS, Sav A, Kelly F (2014). How to attract them and keep them: the pharmacy attributes that matter to Australian residents with chronic conditions. Int J Pharm Pract.

[19] Whitty JA, Kendall E, Sav A (2015). Preferences for the delivery of community pharmacy services to help manage chronic conditions. Res Social Adm Pharm.

[20] McMillan SS, Kelly F, Sav A (2014). Australian community pharmacy services: a survey of what people with chronic conditions and their carers use versus what they consider important. BMJ Open.

[21] Diaz RR, Satheesh G, Moran AE (2026). Delivery models to improve adherence to medicines for chronic diseases in primary care. *The Lancet Primary Care*.

[22] Song HJ, Dennis S, Levesque J-F (2019). What matters to people with chronic conditions when accessing care in Australian general practice? A qualitative study of patient, carer, and provider perspectives. BMC Fam Pract.

[23] Wong MCS, Tam WWS, Wang HHX (2015). Duration of initial antihypertensive prescription and medication adherence: A cohort study among 203,259 newly diagnosed hypertensive patients. Int J Cardiol.

[24] Australian New Zealand Clinical Trials Registry (ANZCTR) (2025). Trial review. https://www.anzctr.org.au/Trial/Registration/TrialReview.aspx?id=390557&isReview=true.

