# Supplementary material for: Patient preferences for antihypertensive medication dispensing trade-offs in cardiovascular disease prevention: national survey in Australia
Source: BMJ Open. 2026 Oct 12;16(10):e122691. doi: 10.1136/bmjopen-2026-122691 (PMC13662037; doi:10.1136/bmjopen-2026-122691)
Supplement: Supplementary file 1 [file bmjopen-2026-122691.reviewer_comments.pdf]

PEER REVIEW HISTORY

BMJ Open publishes all reviews undertaken for accepted manuscripts. Reviewers are asked to complete a checklist review form and are provided with free text boxes to elaborate on their assessment. These free text comments are reproduced below.

ARTICLE DETAILS

Title (Provisional)

Patient preferences for antihypertensive medication dispensing trade-offs in cardiovascular disease prevention: National survey in Australia

Authors

Bonner, Carissa; Fajardo, Michael Anthony; Keast, Rachael; Ganguli, Prerna; Atkins, Emily; Chapman, Niamh; Weir, Kristie; Rodgers, Anthony; Schutte, Aletta E

VERSION 1 - REVIEW

**Reviewer**	**1**
**Name**	**Hueb, Whady**
**Affiliation**	**Universidade Federal de Sao Paulo**
**Date**	**17-Jul-2026**
**COI**	**None**

To the authors:

Major issues:

1- Was the questionnaire based on stated preference or observed behavior?

2- Did the study assess whether the medication dispensing method affected adherence?

3- Was there an assessment of whether the medication dispensing method contributed to tangible benefits?

4- Did the research evaluate elderly individuals with low digital literacy, low socioeconomic status, or those residing in remote regions and/or with cognitive impairments?

5- The study evaluated dispensing duration, delivery methods, and costs; however, it did not assess ease of access to the service, medication waste, polypharmacy, or the patients' clinical stability.

6- The study's results are applicable only to Australia; generalization should be approached with caution.

7- The financial impact on different social groups was not studied.

It was not investigated whether dispensing medication for longer periods affected blood pressure control stability.

8- There was no periodic blood pressure monitoring in this scenario; patients who are newly diagnosed or undergoing therapeutic adjustment may not be the best candidates for long intervals between dispensing events.

9- No cost-effectiveness analyses were conducted, nor were analyses regarding low- and middle-income patients.

10- Did this study include behavioral and psychological analyses, such as health literacy, risk perception, and trust in the healthcare system?

11- This study should have addressed an analysis of medication dispensing policies; logistics models (pharmacy vs. mail delivery); dispensing intervals (1, 2, 3, or 4 months); costs to the patient and the system; and the influence of government subsidies on user preferences.

**Reviewer**	**2**
**Name**	**Labrosciano, Clementine**
**Affiliation**	**The University of Adelaide School of Medicine, Cardiology**
**Date**	**31-Jul-2026**
**COI**	**None**

This national online Australian survey conducted from Nov to Dec 2024 of 2,054 adults taking antihypertensives aimed to investigate predictors of consumer preferences for different antihypertensive dispensing polices given six dispensing options. The authors found that participants preferred short dispensing through pharmacies unless cheaper costs resulted from longer dispensing via post/mail.

Introduction – in the opening paragraph you discuss the global uncontrolled hypertension crisis, it is unclear what the major reason for patients not taking their antihypertensives is.

Methods - This data is now almost 2 years old, is it still relevant?

Figure 1 illustrates the point clearly.

Table 1 – could be presented more scientifically, for example by including p values. The methods doesn’t tell the reader if and how you ran descriptive statistics on your cohort.

Results - How did you choose the 3 age categories? Table 3 is poorly set out.

Discussion - Strengths and limitations section provides sufficent acknowledgments.

A strength of this study is the heavy focus on consumers, with a consumer on the author list and involvement in the co-design of the survey.

Future directions – economic analyses and consumer qualitative. Would you consider measuring medication adherence in future studies?

VERSION 1 - AUTHOR RESPONSE

Reviewer: 1

Thank you for your review. We have mentioned the study limitations you identified, and will investigate additional outcomes and subgroups through a current trial to look at additional outcomes. We have provided answers to each point below.

Major issues:

1- Was the questionnaire based on stated preference or observed behavior?

The survey is based on stated preference. We have removed the word observational to make this clearer where we describe the study design in the strengths and limitations section under the abstract, and method.

Strengths and limitations: The study design was a self reported preference survey…Stated preferences may not reflect real-world behaviour.

Methods: This was an online self reported preference survey of a large national sample of people in Australia taking antihypertensives.

2- Did the study assess whether the medication dispensing method affected adherence?

The survey is focused on self reported dispensing preferences and this study design cannot answer research questions about adherence. Adherence is however an outcome in the trial we are now conducting. We have added this to the discussion.

Discussion: The results have informed the design of a large national trial to test the effect of longer dispensing duration in Australia [25], which will include measures of medication adherence and blood pressure control.

3- Was there an assessment of whether the medication dispensing method contributed to tangible benefits?

As above, the focus of the survey was on dispensing preferences only and the predictors of this. The trial will look at tangible benefits in terms of blood pressure control and medication adherence.

Discussion: The results have informed the design of a large national trial to test the effect of longer dispensing duration in Australia [25], which will include measures of medication adherence and blood pressure control.

4- Did the research evaluate elderly individuals with low digital literacy, low socioeconomic status, or those residing in remote regions and/or with cognitive impairments?

The quota sampling included consideration of age and education (which is correlated with heath literacy) to ensure diversity on these outcomes. Regionality is provided in the participant demographics. We did not assess cognitive impairment as that was not a focus of the research question. We have explained the variables in methods with full details in Supplementary Materials.

Methods: Logistic regressions were conducted for each choice set to assess predictors of preferences for options in each choice set. This included the following categorical binary predictors: whether the PBS safety threshold was reached, whether participants lived in a major city (versus not), preference for 1 to 2 months dispensing (versus 3–6 months), preference for pharmacy pick up options (versus delivery), university education (versus no university education), female (versus male), under 65 year olds (versus 65 years and over) and weekly income as a continuous variable.

5- The study evaluated dispensing duration, delivery methods, and costs; however, it did not assess ease of access to the service, medication waste, polypharmacy, or the patients' clinical stability.

This is correct. However, some of these issues did arise in open response comments, which we will report in a separate paper following content analysis. This is mentioned in the

discussion.

Discussion: The consumer advisors will be involved in coding open responses about dispensing preferences, for a future qualitative paper on the reasons for different preferences.

6- The study's results are applicable only to Australia; generalization should be approached with caution.

We agree this is a limitation and have stated this in the strengths and limitations section under the abstract, and have now added it to the discussion as well. We have also modified the title to make this clearer.

Title: Patient preferences for antihypertensive medication dispensing trade-offs in cardiovascular disease prevention: National survey in Australia

Strengths and limitations: The financial context in Australia may not generalise to other countries.

Discussion: Finally, the decisions individuals make in a vignette may not fully reflect their real-life choices, and the financial context in Australia may not generalise to other countries.

7- The financial impact on different social groups was not studied.

We have included financial measures in the analyses, but we did not plan subgroup analyses of different social groups.

8 - It was not investigated whether dispensing medication for longer periods affected blood pressure control stability.

We agree the survey cannot answer this question but will investigate this outcome in the trial as now stated:

Discussion: The results have informed the design of a large national trial to test the effect of longer dispensing duration in Australia [25], which will include measures of medication

adherence and blood pressure control.

9 - There was no periodic blood pressure monitoring in this scenario; patients who are newly diagnosed or undergoing therapeutic adjustment may not be the best candidates for long intervals between dispensing events.

We agree and have considered this in trial eligibility.

10 - No cost-effectiveness analyses were conducted, nor were analyses regarding low- and middle-income patients.

This will be investigated in the trial we are currently running.

11 - Did this study include behavioral and psychological analyses, such as health literacy, risk perception, and trust in the healthcare system?

We did not include these measures in the survey as they do not relate to the research question we were focused on, and the consumer advisors preferred to keep the survey shorted given the large number of complex forced choice questions. However we have extensively investigated these issues in related but separate projects.

12 - This study should have addressed an analysis of medication dispensing policies; logistics models (pharmacy vs. mail delivery); dispensing intervals (1, 2, 3, or 4 months); costs to the patient and the system; and the influence of government subsidies on user preferences.

We agree that additional research could investigate additional dispensing policies and costs from different perspectives, and have provided referenced papers on our team's broader work (e.g. Wang et al. MJA 2026). For the purpose of this study, the variables cover some but not all of your suggestions. We have explained the variables in methods with full details in Supplementary Materials.

Methods: Logistic regressions were conducted for each choice set to assess predictors of preferences for options in each choice set. This included the following categorical binary predictors: whether the PBS safety threshold was reached, whether participants lived in a

major city (versus not), preference for 1 to 2 months dispensing (versus 3–6 months), preference for pharmacy pick up options (versus delivery), university education (versus no university education), female (versus male), under 65 year olds (versus 65 years and over) and weekly income as a continuous variable.

Reviewer: 2

Thank you for your review. We have clarified the tables and have provided answers to each point below.

Comments to the Author:
This national online Australian survey conducted from Nov to Dec 2024 of 2,054 adults taking antihypertensives aimed to investigate predictors of consumer preferences for different antihypertensive dispensing polices given six dispensing options. The authors found that participants preferred short dispensing through pharmacies unless cheaper costs resulted from longer dispensing via post/mail.

Introduction – in the opening paragraph you discuss the global uncontrolled hypertension crisis, it is unclear what the major reason for patients not taking their antihypertensives is.

We have focused on the issue of dispensing as a behavioural driver/barrier for the purpose of this paper as that was the key research question. However we acknowledge there other reasons for non-adherence and have now mentioned this in the discussion.

Introduction: [2]. The World Health Organisation has identified access to antihypertensives as the cornerstone of hypertension control [6]….A key component of access to antihypertensives is dispensing duration…Longer dispensing durations may improve adherence by reducing the burden on the patient to refill their medications frequently; and reduced dispensing fees may be associated with lower costs to patients and the healthcare system [5]. A large retrospective US claims analysis found 90-day scripts were associated with a 27% improvement in adherence [7, 8]. In Thailand, changing from a 30-day to 90-day dispensing duration improved adherence to statins and diabetes medication [9].

Discussion: This study focuses on opportunity barriers to adherence in terms of costs and ease of access. Additional interventions would be needed to address motivational barriers to adherence, such as experience of side effects or low perceived risk.

Methods - This data is now almost 2 years old, is it still relevant?

We confirm the policy context has not changed since we conducted the study, and have included a recent reference that demonstrates this (Wang et al. MJA 2026).

Figure 1 illustrates the point clearly.

Thank you for this comment.

Table 1 – could be presented more scientifically, for example by including p values. The methods doesn't tell the reader if and how you ran descriptive statistics on your cohort.

Table 1 provides a description of the forced choice responses in the methods section. Tables 3-4 provide p values for the statistical models in the results section. We have added descriptive statistics to the method section.

Methods: Descriptive statistics to describe sample characteristics were calculated using frequencies and means.

Results - How did you choose the 3 age categories? Table 3 is poorly set out.

The three age categories were used for quota sampling to ensure adequate representation from a range of ages, as we anticipated that dispensing preferences may vary by age. However this was converted to a binary outcome for analyses on statistical grounds. We have explained the approach to age in the methods. We have tried multiple versions of Table 3 and acknowledge it is complex due to the study design with multiple forced choices and predictors. However this was the most simplified format for the table that we were able to produce for the complex design. We have now added alternate shading to make the rows easier to follow in both Table 1 and Tables 3-4; and would be happy to revise the formatting further if there is a specific

suggestion.

Methods: Quota sampling was used to ensure sufficient diversity in age, gender and education groups…Checks of model fit between age in years versus age groups (18-44, 45-65, 65+) showed marginal changes in model fit statistics (-2LL, pseudo R2) across models despite violating linearity assumption, thus for ease of interpretation age was treated as a binary categorical variable in all models. The cut point for age was guided by policy decisions as 65 years is an important change in access to medications and screening decisions.

Results: See formatting changes in Tables 3-4, which also include the p values requested above.

Discussion - Strengths and limitations section provides sufficent acknowledgments.
A strength of this study is the heavy focus on consumers, with a consumer on the author list and involvement in the co-design of the survey.

Thank you for this comment. We have added some additional limitations to address reviewer 1's comments. We have also included a heading for consumer involvement as requested by the editor.

Future directions – economic analyses and consumer qualitative. Would you consider measuring medication adherence in future studies?

This is the focus of a current trial investigating whether alternative blood pressure dispensing policies can improve blood pressure control via increased adherence. We have clarified this in the discussion to address both reviewers' comments on this.

Discussion: The results have informed the design of a large national trial to test the effect of longer dispensing duration in Australia [25], which will include measures of medication adherence and blood pressure control.
