# Supplementary material for: Patient preferences for antihypertensive medication dispensing trade-offs in cardiovascular disease prevention: national survey in Australia
Source: BMJ Open. 2026 Oct 12;16(10):e122691. doi: 10.1136/bmjopen-2026-122691 (PMC13662037; doi:10.1136/bmjopen-2026-122691)
Supplement: Supplementary file 2 [file bmjopen-16-10-s001.docx]

# **Supplementary 1: Survey process and questions**

The author team and Dynata separately tested the functionality of the survey prior to launch for both web and mobile screens. There was a total of 26 screens for participants to complete. Screens included participation information, section breaks or survey questions. At most, a screen would include four questions. Responses were considered completed if respondents got to the last ‘Thank You’ screen. Not all questions were required, which contributed to missing data. Respondents could not go back and review their responses. Potential duplicate responses were controlled for via Dynata as part of their quality assurance processes.

Initially, respondents were provided with the Participant Information Statement which described that data was collected anonymously, the estimated length of the survey, investigator information and purpose of the study to help them decide whether they wanted to consent to the study. If they consented, they were taken to the survey (available in the appendix). Respondents first completed quota and eligibility questions and depending on these response and quotas, respondents were either taken to the survey or thanked for their interest following Dynata procedures. Respondents taken to the survey then completed three sections sequentially with no randomisation or alternation of question presentation. Section 1 were ‘About You’ questions which included: concession card holder status, whether respondent reached Pharmaceutical Benefits Scheme Safety Net in a calendar year, amount of different medications (prescribed and not prescribed) taken per week, whether their current medication collection was typically via post or picked up at the pharmacy, self-reported blood pressure information, perceptions of costs of medicine. In Australia, the Pharmaceutical Benefits Scheme (PBS) Safety Net is the threshold up to which patients pay full cost of medications within a calendar year. In 2024, the threshold was $277.20 AUD for concession card holders and $1647.90 AUD for non-concession card holders and are annually updated to align with the Consumer Price Index on January 1. Patients are also allowed to apply for a PBS Safety Net Card if they exceed this threshold to continue accessing cheaper medicines within the same calendar year. For section 1, an average of four questions were presented per screen.

Section 2 included questions about respondent’s preferences about getting medicine. Questions included: preferred delivery format (pharmacy pickup or delivery), medication dispensing amount (1 to 6 months), questions about willingness to participate in a hypothetical randomised controlled trial and forced-choice options depending on concession card holder status. An example of this is shown in Figure 1. If a respondent indicated ‘Don’t Know’ to concession card holder status, they were considered as a non-concession card holder for this part of the survey. The separation was important because in Australia concession card holders can purchase medications at a lower price. Costs used were presented based on PBS prices in May 2024. Respondents viewed one forced choice set at a time (i.e. they were not presented simultaneously).

Section 3 included questions about respondents’ experience with antihypertensive medication. Questions asked respondents to indicate how many antihypertensive medications they took per day, how long their medication supply typically lasted, costs of medicines, number of repeat prescriptions and how repeat prescriptions were collected.

## Online Survey Questions

**Please answer the following questions to determine if you are eligible to take part.**

Q. Do you live in Australia?

- Yes
- No

Q. What is your age category?

- Under 18 years
- 18 to 44 years
- 45 to 64 years
- 65 years or over

Q. In the last two weeks, have you taken any medicine for high blood pressure?

- Yes
- No
- Don’t know

**Quota sampling**

Q. What is your year of birth? ______

Q. What is your highest level of education?

- Below Year 10 (including Certificate I/II)
- Year 10 or equivalent (e.g. Leaving Certificate)
- Year 11
- Year 12 or equivalent (e.g. HSC, VCE)
- Certificate III/IV
- Diploma/Associate degree
- University degree (e.g. Bachelor degree)
- Graduate diploma or graduate certificate
- Postgraduate (e.g. Masters)

**Section 1 of 3: About you**

**This section will help us work out if different groups have different preferences.**

Q. Are you a concession card holder?

- Yes
- No
- Don’t know

Q. Do you usually reach the Pharmaceutical Benefits Scheme (PBS) Safety Net in a calendar year?

- Yes
- No
- Don’t know

Q. How many different kinds of medicines do you take in a typical week (please include all types of medicines, not just blood pressure medicines)?

Number of prescription medicines:

- 0
- 1-3
- 4-6
- 7-10
- 11-20
- More than 20

Number of non-prescription medicines (also referred to as over-the-counter medicines) or herbal/natural supplements:

- 0
- 1-3
- 4-6
- 7-10
- 11-20
- More than 20

Q. Which method do you use most frequently to get your medicines?

- I receive medicines via postal or courier delivery
- I pick up medicines in-person at a pharmacy

Q. Please rate how much you agree with the following statement: *I spend a lot of money on my medicines.*

- Strongly agree
- Agree
- Don’t know
- Disagree
- Strongly disagree

Q. In the last 12 months, have you or a health professional measured your blood pressure?

- Yes, I have measured my blood pressure
- Yes, a health professional has measured my blood pressure
- All of the above
- None of the above
- Don’t know

Q. Has a health professional (e.g. doctor, nurse) ever told you that you have high blood pressure (hypertension)?

- Yes
- No
- Don’t know

Q. [If yes above] When you/your health professional measure your blood pressure, what is your reading typically?

Systolic blood pressure (larger top number)

- Less than or equal to 120
- 121 - 129
- 130 - 144
- More than or equal to 145
- Not sure

Diastolic blood pressure (smaller bottom number)

- Less than or equal to 80
- 81 - 84
- 85 - 89
- More than or equal to 90
- Not sure

**Section 2 of 3: Your preferences** 

**These questions will help us understand your preferences about getting medicine.**

Q. Imagine your doctor has told you to take a new blood pressure medicine for the next year. They offer you some choices for how to get the medicine.

What is your preferred delivery format?

- Pick up at a pharmacy
- Delivery by post or courier

Reasons for this choice: _________________________

If you prefer delivery, what is the most you would pay for this?

- $0
- $1-5
- $6-10
- $11-15
- $16-20

How many months’ worth of medicine would you prefer to get at a time?

- 1 month
- 2 months
- 3 months
- 4 months

Reasons for this choice: _________________________

Q. Now imagine your doctor says they are participating in a research study to see which option helps people manage their blood pressure the best. It is all about how you receive the medicines your doctor prescribes. All costs will be covered, and you can still see your GP as often as you want. If you choose to participate, you would receive your medicine in one of the following ways. The option will be chosen for you at random. You can’t change which option you will get.

- Pick-up at your usual pharmacy, 1 month supply
- Pick-up at your usual pharmacy, 2 month supply
- Pick-up at your usual pharmacy, 3 month supply
- Postal delivery, 1 month supply
- Postal delivery, 2 month supply
- Postal delivery, 3 month supply
- Postal delivery, 4 month supply

Would you participate in this research study?

- Yes
- No

Q. Please explain the reason for your response: _____________________________

**DIFFERENT COST-BASED BLOCKS: 6 pairwise comparisons with different costs shown for concession vs non-concession; questions shown are determined by concession card status (‘Don’t Know’ responses are considered as ‘non-concession card’).**

Now imagine your doctor offers you different options for getting your blood pressure medicine.

1. Which of these options would you prefer? (Choice Set 1: Delivery difference 2-months)

| Non-Concession Card Holder   | Concession Card Holder   |
| --- | --- |

2. Which of these options would you prefer? (Choice Set 2: Delivery Difference, 3 months)

| Non-Concession Card Holder | Concession Card Holder   |
| --- | --- |

3. Which of these options would you prefer? (Choice Set 3: Pharmacy pick up, 2 v 3 months)

| Non-Concession Card Holder   | Concession Card Holder   |
| --- | --- |

4. Which of these options would you prefer? (Choice Set 4: Delivery 1 v 4 months)

| Non Concession Card Holder   | Concession Card Holder   |
| --- | --- |

5. Which of these options would you prefer? (Choice Set 5: Pharmacy Pick up 1 vs 4 months)

| Non Concession Card Holder   | Concession Card Holder   |
| --- | --- |

6. Which of these options would you prefer? (Choice Set 6: Delivery, 2 v 3 months)

| Non Concession Card Holder   | Concession Card Holder   |
| --- | --- |

**Non-Concession Card Holder Questions:**

Q Please indicate how affordable each of these costs would be for you to pay at once for medicine [Likert scale 1 not affordable to 5 completely affordable]:

- $15.90 for 1 month supply
- $18.40 for 2 month supply
- $20.90 for 3 month supply
- $23.40 for 4 month supply

Q Please indicate how affordable each of these costs would be for you to pay in a year for medicine: [Likert scale 1 not affordable to 5 completely affordable]:

- $70.20 per year
- $83.60 per year
- $110.40 per year
- $190.80 per year

**Concession Card Holder Questions:**

Q Please indicate how affordable each of these costs would be for you to pay at once for medicine [Likert scale 1 not affordable to 5 completely affordable]:

- $7.70 for 1 month supply
- $7.70 for 2 month supply
- $7.70 for 3 month supply
- $7.70 for 4 month supply

Q Please indicate how affordable each of these costs would be for you to pay in a year for medicine: [Likert scale 1 not affordable to 5 completely affordable]:

- $23.10 per year
- $30.80 per year
- $46.20 per year
- $92.80 per year

**Section 3 of 3: Your experience**

**This section is about your experience with blood pressure medicines.**

Q. Please list all the names of all the medicines you take regularly: ______

Q. How many different **blood pressure** medicines do you take per day? We are interested in how many different *types* of blood pressure medicines you take each day, not the number of tablets you take (determines how many times the following questions are shown)

- 1
- 2
- 3
- 4
- 5
- More than 5
- Not sure

Q. Each time you pick up your blood pressure medicine, how long does it usually last?

- 30 days
- 60 days
- Other, please specify: _________________________
- Not sure

Q. How much do you usually pay each time you pick up your blood pressure medicine (after Medicare rebate or other subsidies)?

- Less than $8
- $8 - $14.99
- $15 - $17.99
- $18 - $22.99
- $23 - $27.99
- $28 - $31.99
- $32 or more
- Not sure

Q. How many repeats are usually on the prescription for your blood pressure medicine?

- Please specify: ________________
- Don’t know

Q. To get a new prescription, who do you need to see?

- GP – in person
- GP – online or phone consultation
- Nurse
- Pharmacist
- Cardiologist
- Other: ____________________

Q. How much do you usually pay out of pocket for the appointment to get a new prescription? ___

## **Supplementary 2: Statistical Tables**

Table S1: Details per choice set and frequencies for selected options for concession card and non-concession card holders (bolded cells indicate the preferred option within each set)

|  | **Concession Card Holders** | |  | **Non-concession Card Holders** | |
| --- | --- | --- | --- | --- | --- |
| **Choice Set** | **Option A** | **Option B** |  | **Option A** | **Option B** |
| 1: Pharmacy vs Postal, both 60 days | **1100 (82.5%)**  **Pharmacy Pickup**  **60 pills**  **$7.70/order**  **$46.20/year** | 233 (17.5%)  Postal Delivery  60 pills  $7.70/order  $46.20/year |  | **543 (76.5%)**  **Pharmacy Pickup**  **60 pills**  **$18.40/order**  **$110.40/year** | 167 (23.5%)  Postal Delivery  60 pills  $18.40/order  $110.40/year |
| 2: Pharmacy vs Postal, both 90 days | **1060 (79.5%)**  **Pharmacy Pickup**  **90 pills**  **$7.70/order**  **$30.80/year** | 274 (20.5%)  Postal Delivery  90 pills  $7.70/order  $30.80/year |  | **533 (75.0%)**  **Pharmacy Pickup**  **90 pills**  **$20.90/order**  **$83.60/year** | 178 (25.%)  Postal Delivery  90 pills  $20.90/order  $83.60/year |
| 3: Pharmacy, 60 vs 90 days | 416 (31.2%)  Pharmacy Pickup  60 pills  $7.70/order  $46.20/year | **917 (68.8%)**  **Pharmacy Pickup**  **90 pills**  **$7.70/order**  **$30.80/year** |  | 135 (19.0%)  Pharmacy Pickup  60 pills  $18.40/order  $110.40/year | **576 (81.0%)**  **Pharmacy Pickup**  **90 pills**  **$20.90/order**  **$83.60/year** |
| 4: Postal, 30 vs 120 days | 220 (16.5%)  Postal Delivery  30 pills  $7.70/order  $92.40/year | **1115 (83.5%)**  **Postal Delivery**  **120 pills**  **$7.70/order**  **$23.10/year** |  | 59 (8.3%)  Postal Delivery  30 pills  $15.90/order  $190.80/year | **651 (91.7%)**  **Postal Delivery**  **120 pills**  **$23.40/order**  **$70.20/year** |
| 5: Pharmacy, 30 vs 120 days | 213 (16.0%)  Pharmacy Pickup  30 pills  $7.70/order  $92.40/year | **1119 (84.0%)**  **Pharmacy Pickup**  **120 pills**  **$7.70/order**  **$23.10/year** |  | 57 (8.0%)  Pharmacy Pickup  30 pills  $15.90/order  $190.80/year | **652 (92.0%)**  **Pharmacy Pickup**  **120 pills**  **$23.40/order**  **$70.20/year** |
| 6: Postal, 60 vs 90 days | 214 (16.1%)  Postal Delivery  60 pills  $7.70/order  $46.20/year | **1117 (83.9%)**  **Postal Delivery**  **90 pills**  **$7.70/order**  **$30.80/year** |  | 58 (8.2%)  Postal Delivery  60 pills  $18.40/order  $110.40/year | **651 (91.8%)**  **Postal Delivery**  **90 pills**  **$20.90/order**  **$83.60/year** |
| *Note: For Concession Card Holders Choice 1 and Choice 3 missing data n=2, Choice 2 missing data n=1, Choice 5 missing n = 3, Choice 6 missing data n=4. For non-concession card holders: Note: Choice 1 missing data n=1, Choice 4 missing data n=1, Choice 5 and Choice 6 missing data n=2.* | | | | | |

## Logistic Regression Models: Parameter Estimates and Model Summary Statistics

### Model 1.1 Outcome: Preferred Option for Choice Set 1 for Concession Card Holders (1=Option A, 0=Option B), N=1187

| **Binary Predictor Level of Interest** | **Reference Category** | **B** | **S.E.** | **Wald** | **df** | **Sig.** | **OR** | **95% CI for OR** | |
| --- | --- | --- | --- | --- | --- | --- | --- | --- | --- |
|  |  |  |  |  |  |  |  | **Lower** | **Upper** |
| Reached PBS Safety Net Threshold | Did not reach threshold | .097 | .230 | .176 | 1 | .675 | 1.101 | .702 | 1.729 |
| Lives in major city | Does not live in major city | .025 | .234 | .011 | 1 | .915 | 1.025 | .648 | 1.622 |
| Prefers 1–2 months delivery | Prefers 3–6 months | .858 | .190 | 20.339 | 1 | <.001 | 2.357 | 1.624 | 3.422 |
| Prefers Pharmacy Pickup | Prefers Delivery | 2.993 | .229 | 171.075 | 1 | <.001 | 19.940 | 12.734 | 31.224 |
| University educated | Not university educated | .033 | .221 | .022 | 1 | .882 | 1.033 | .671 | 1.592 |
| Female | Male | -.050 | .191 | .067 | 1 | .795 | .952 | .654 | 1.384 |
| Under 65 Years | 65 years and over | -.443 | .198 | 5.041 | 1 | .025 | .642 | .436 | .945 |
| **Continuous Predictor** |  |  |  |  |  |  |  |  |  |
| Income |  | .013 | .035 | .141 | 1 | .707 | 1.013 | .947 | 1.084 |
| **Constant** |  | -1.185 | .467 | 6.434 | 1 | .011 | .306 |  |  |
| Notes: For gender, the three responses that were neither male nor female were excluded listwise from this analysis. Weekly income was treated as continuous given the ordinal nature of its collection and spread of results. Discrete continuous categories were: No income, $1–149, $150–299, $300–399, $400–499, $500–649, $650–799, $800–999, $1000–1249, $1250–1499, $1500–1749, $1750–1999, $2000–2999 and $3000+. | | | | | | | | | |

| **Model Summary** | | | |
| --- | --- | --- | --- |
|  | -2 Log likelihood | Cox & Snell R Square | Nagelkerke R Square |
|  | 787.539 | .231 | .382 |

| **Hosmer and Lemeshow Test** | | |
| --- | --- | --- |
| Chi-square | df | Sig. |
| 19.226 | 8 | .014 |

### Model 1.2 Outcome: Preferred Option for Choice Set 2 for Concession Card Holders (1=Option A, 0=Option B),N=1188

| **Binary Predictor Level of Interest** | **Reference Category** | **B** | **S.E.** | **Wald** | **df** | **Sig.** | **OR** | **95% CI for OR** | |
| --- | --- | --- | --- | --- | --- | --- | --- | --- | --- |
|  |  |  |  |  |  |  |  | **Lower** | **Upper** |
| Reached PBS Safety Net Threshold | Did not reach threshold | .112 | .208 | .291 | 1 | .589 | 1.119 | .744 | 1.683 |
| Lives in major city | Does not live in major city | -.037 | .214 | .029 | 1 | .864 | .964 | .634 | 1.467 |
| Prefers 1–2 months delivery | Prefers 3–6 months | .760 | .175 | 18.911 | 1 | <.001 | 2.139 | 1.518 | 3.013 |
| Prefers Pharmacy Pickup | Prefers Delivery | 2.614 | .218 | 143.613 | 1 | <.001 | 13.650 | 8.902 | 20.931 |
| University educated | Not university educated | -.240 | .198 | 1.477 | 1 | .224 | .787 | .534 | 1.158 |
| Female | Male | -.021 | .175 | .014 | 1 | .906 | .980 | .695 | 1.380 |
| Under 65 Years | 65 years and over | -.882 | .180 | 24.026 | 1 | <.001 | .414 | .291 | .589 |
| **Continuous Variable** |  |  |  |  |  |  |  |  |  |
| Income |  | .045 | .032 | 2.033 | 1 | .154 | 1.046 | .983 | 1.113 |
| **Constant** |  | -1.014 | .431 | 5.525 | 1 | .019 | .363 |  |  |
| Notes: For gender, the three responses that were neither male nor female were excluded listwise from this analysis. Weekly income was treated as continuous given the ordinal nature of its collection and spread of results. Discrete continuous categories were: No income, $1–149, $150–299, $300–399, $400–499, $500–649, $650–799, $800–999, $1000–1249, $1250–1499, $1500–1749, $1750–1999, $2000–2999 and $3000+. | | | | | | | | | |

| **Model Summary** | | | |
| --- | --- | --- | --- |
|  | -2 Log likelihood | Cox & Snell R Square | Nagelkerke R Square |
|  | 901.273 | .227 | .355 |

| **Hosmer and Lemeshow Test** | | |
| --- | --- | --- |
| Chi-square | df | Sig. |
| 8.875 | 8 | .353 |

### Model 1.3 Outcome: Preferred Option for Choice Set 3 or Concession Card Holders (1=Option A, 0=Option B), N=1188

| **Binary Predictor Level of Interest** | **Reference Category** | **B** | **S.E.** | **Wald** | **df** | **Sig.** | **OR** | **95% CI for OR** | |
| --- | --- | --- | --- | --- | --- | --- | --- | --- | --- |
|  |  |  |  |  |  |  |  | **Lower** | **Upper** |
| Reached PBS Safety Net Threshold | Did not reach threshold | .969 | .172 | 31.679 | 1 | <.001 | 2.635 | 1.880 | 3.692 |
| Lives in major city | Does not live in major city | .167 | .156 | 1.158 | 1 | .282 | 1.182 | .871 | 1.604 |
| Prefers 1–2 months delivery | Prefers 3–6 months | -.010 | .141 | .005 | 1 | .944 | .990 | .751 | 1.306 |
| Prefers Pharmacy Pickup | Prefers Delivery | .100 | .195 | .263 | 1 | .608 | 1.105 | .755 | 1.618 |
| University educated | Not university educated | .403 | .146 | 7.644 | 1 | .006 | 1.496 | 1.124 | 1.990 |
| Female | Male | -.244 | .131 | 3.492 | 1 | .062 | .783 | .606 | 1.012 |
| Under 65 Years | 65 years and over | .096 | .137 | .490 | 1 | .484 | 1.100 | .842 | 1.439 |
| **Continuous Variable** |  |  |  |  |  |  |  |  |  |
| Income |  | -.007 | .024 | .077 | 1 | .782 | .993 | .948 | 1.041 |
| **Constant** |  | -1.794 | .356 | 25.462 | 1 | <.001 | .166 |  |  |
| Notes: For gender, the three responses that were neither male nor female were excluded listwise from this analysis. Weekly income was treated as continuous given the ordinal nature of its collection and spread of results. Discrete continuous categories were: No income, $1–149, $150–299, $300–399, $400–499, $500–649, $650–799, $800–999, $1000–1249, $1250–1499, $1500–1749, $1750–1999, $2000–2999 and $3000+. | | | | | | | | | |

| **Model Summary** | | | |
| --- | --- | --- | --- |
|  | -2 Log likelihood | Cox & Snell R Square | Nagelkerke R Square |
|  | 1421.184^a^ | .047 | .066 |

| **Hosmer and Lemeshow Test** | | |
| --- | --- | --- |
| Chi-square | df | Sig. |
| 5.598 | 8 | .692 |

### Model 1.4 Outcome: Preferred Option for Choice Set 4 for Concession Card Holders (1=Option A, 0=Option B), N=1189

| **Binary Predictor Level of Interest** | **Reference Category** | **B** | **S.E.** | **Wald** | **df** | **Sig.** | **OR** | **95% CI for OR** | |
| --- | --- | --- | --- | --- | --- | --- | --- | --- | --- |
|  |  |  |  |  |  |  |  | **Lower** | **Upper** |
| Reached PBS Safety Net Threshold | Did not reach threshold | .824 | .253 | 10.592 | 1 | .001 | 2.279 | 1.388 | 3.743 |
| Lives in major city | Does not live in major city | .708 | .251 | 7.976 | 1 | .005 | 2.030 | 1.242 | 3.317 |
| Prefers 1–2 months delivery | Prefers 3–6 months | -.328 | .175 | 3.510 | 1 | .061 | .720 | .511 | 1.015 |
| Prefers Pharmacy Pickup | Prefers Delivery | -.493 | .210 | 5.523 | 1 | .019 | .611 | .405 | .921 |
| University educated | Not university educated | .656 | .196 | 11.165 | 1 | <.001 | 1.927 | 1.311 | 2.830 |
| Female | Male | -.076 | .172 | .194 | 1 | .660 | .927 | .662 | 1.298 |
| Under 65 Years | 65 years and over | .732 | .181 | 16.335 | 1 | <.001 | 2.079 | 1.458 | 2.965 |
| **Continuous Variable** |  |  |  |  |  |  |  |  |  |
| Income |  | .082 | .031 | 6.900 | 1 | .009 | 1.085 | 1.021 | 1.153 |
| **Constant** |  | -3.709 | .476 | 60.603 | 1 | <.001 | .024 |  |  |
| Notes: For gender, the three responses that were neither male nor female were excluded listwise from this analysis. Weekly income was treated as continuous given the ordinal nature of its collection and spread of results. Discrete continuous categories were: No income, $1–149, $150–299, $300–399, $400–499, $500–649, $650–799, $800–999, $1000–1249, $1250–1499, $1500–1749, $1750–1999, $2000–2999 and $3000+. | | | | | | | | | |

| **Model Summary** | | | |
| --- | --- | --- | --- |
|  | -2 Log likelihood | Cox & Snell R Square | Nagelkerke R Square |
|  | 919.024^a^ | .120 | .202 |

| **Hosmer and Lemeshow Test** | | |
| --- | --- | --- |
| Chi-square | df | Sig. |
| 13.103 | 8 | .108 |

### Model 1.5 Outcome: Preferred Option for Choice Set 5 for Concession Card Holders (1=Option A, 0=Option B), N=1186

| **Binary Predictor Level of Interest** | **Reference Category** | **B** | **S.E.** | **Wald** | **df** | **Sig.** | **OR** | **95% CI for OR** | |
| --- | --- | --- | --- | --- | --- | --- | --- | --- | --- |
|  |  |  |  |  |  |  |  | **Lower** | **Upper** |
| Reached PBS Safety Net Threshold | Did not reach threshold | .828 | .256 | 10.468 | 1 | .001 | 2.289 | 1.386 | 3.781 |
| Lives in major city | Does not live in major city | .647 | .249 | 6.756 | 1 | .009 | 1.910 | 1.172 | 3.110 |
| Prefers 1–2 months delivery | Prefers 3–6 months | -.315 | .177 | 3.158 | 1 | .076 | .730 | .516 | 1.033 |
| Prefers Pharmacy Pickup | Prefers Delivery | -.334 | .216 | 2.392 | 1 | .122 | .716 | .469 | 1.093 |
| University educated | Not university educated | .756 | .198 | 14.554 | 1 | <.001 | 2.130 | 1.444 | 3.142 |
| Female | Male | -.588 | .176 | 11.130 | 1 | <.001 | .555 | .393 | .785 |
| Under 65 Years | 65 years and over | .968 | .185 | 27.361 | 1 | <.001 | 2.632 | 1.832 | 3.783 |
| **Continuous Variable** |  |  |  |  |  |  |  |  |  |
| Income |  | .068 | .031 | 4.644 | 1 | .031 | 1.070 | 1.006 | 1.138 |
| **Constant** |  | -3.645 | .484 | 56.648 | 1 | <.001 | .026 |  |  |
| Notes: For gender, the three responses that were neither male nor female were excluded listwise from this analysis. Weekly income was treated as continuous given the ordinal nature of its collection and spread of results. Discrete continuous categories were: No income, $1–149, $150–299, $300–399, $400–499, $500–649, $650–799, $800–999, $1000–1249, $1250–1499, $1500–1749, $1750–1999, $2000–2999 and $3000+. | | | | | | | | | |

| **Model Summary** | | | |
| --- | --- | --- | --- |
|  | -2 Log likelihood | Cox & Snell R Square | Nagelkerke R Square |
|  | 897.229^a^ | .126 | .214 |

| **Hosmer and Lemeshow Test** | | |
| --- | --- | --- |
| Chi-square | df | Sig. |
| 10.659 | 8 | .222 |

### Model 1.6 Outcome: Preferred Option for Choice Set 6 for Concession Card Holders (1=Option A, 0=Option B), N=1188

| **Binary Predictor Level of Interest** | **Reference Category** | **B** | **S.E.** | **Wald** | **df** | **Sig.** | **OR** | **95% CI for OR** | |
| --- | --- | --- | --- | --- | --- | --- | --- | --- | --- |
|  |  |  |  |  |  |  |  | **Lower** | **Upper** |
| Reached PBS Safety Net Threshold | Did not reach threshold | 1.459 | .317 | 21.135 | 1 | <.001 | 4.303 | 2.310 | 8.015 |
| Lives in major city | Does not live in major city | .765 | .264 | 8.404 | 1 | .004 | 2.150 | 1.281 | 3.606 |
| Prefers 1–2 months delivery | Prefers 3–6 months | -.237 | .182 | 1.693 | 1 | .193 | .789 | .553 | 1.127 |
| Prefers Pharmacy Pickup | Prefers Delivery | -.625 | .213 | 8.607 | 1 | .003 | .535 | .353 | .813 |
| University educated | Not university educated | .687 | .203 | 11.394 | 1 | <.001 | 1.987 | 1.334 | 2.960 |
| Female | Male | -.439 | .180 | 5.985 | 1 | .014 | .644 | .453 | .916 |
| Under 65 Years | 65 years and over | .922 | .190 | 23.682 | 1 | <.001 | 2.515 | 1.735 | 3.646 |
| **Continuous Variable** |  |  |  |  |  |  |  |  |  |
| Income |  | .111 | .032 | 11.688 | 1 | <.001 | 1.117 | 1.048 | 1.190 |
| **Constant** |  | -4.526 | .532 | 72.273 | 1 | <.001 | .011 |  |  |
| Notes: For gender, the three responses that were neither male nor female were excluded listwise from this analysis. Weekly income was treated as continuous given the ordinal nature of its collection and spread of results. Discrete continuous categories were: No income, $1–149, $150–299, $300–399, $400–499, $500–649, $650–799, $800–999, $1000–1249, $1250–1499, $1500–1749, $1750–1999, $2000–2999 and $3000+. | | | | | | | | | |

| **Model Summary** | | | |
| --- | --- | --- | --- |
|  | -2 Log likelihood | Cox & Snell R Square | Nagelkerke R Square |
|  | 857.852^a^ | .164 | .276 |

| **Hosmer and Lemeshow Test** | | |
| --- | --- | --- |
| Chi-square | df | Sig. |
| 20.130 | 8 | .010 |

### Model 2.1 Outcome: Preferred Option for Choice Set 1 for Non-Concession Card Holders (1=Option A, 0=Option B), N=599

| **Binary Predictor Level of Interest** | **Reference Category** | **B** | **S.E.** | **Wald** | **df** | **Sig.** | **OR** | **95% CI for OR** | |
| --- | --- | --- | --- | --- | --- | --- | --- | --- | --- |
|  |  |  |  |  |  |  |  | **Lower** | **Upper** |
| Reached PBS Safety Net Threshold | Did not reach threshold | .333 | .285 | 1.366 | 1 | .242 | 1.396 | .798 | 2.440 |
| Lives in major city | Does not live in major city | .022 | .299 | .006 | 1 | .941 | 1.022 | .569 | 1.838 |
| Prefers 1–2 months delivery | Prefers 3–6 months | .805 | .215 | 13.998 | 1 | <.001 | 2.236 | 1.467 | 3.409 |
| Prefers Pharmacy Pickup | Prefers Delivery | 2.776 | .378 | 53.866 | 1 | <.001 | 16.055 | 7.650 | 33.695 |
| University educated | Not university educated | .012 | .228 | .003 | 1 | .958 | 1.012 | .648 | 1.581 |
| Female | Male | -.288 | .217 | 1.758 | 1 | .185 | .750 | .490 | 1.148 |
| Under 65 Years | 65 years and over | -.499 | .278 | 3.216 | 1 | .073 | .607 | .352 | 1.047 |
| **Continuous Variable** |  |  |  |  |  |  |  |  |  |
| Income |  | -.126 | .043 | 8.723 | 1 | .003 | .881 | .810 | .958 |
| **Constant** |  | .098 | .659 | .022 | 1 | .882 | 1.103 |  |  |
| Notes: For gender, the three responses that were neither male nor female were excluded listwise from this analysis. Weekly income was treated as continuous given the ordinal nature of its collection and spread of results. Discrete continuous categories were: No income, $1–149, $150–299, $300–399, $400–499, $500–649, $650–799, $800–999, $1000–1249, $1250–1499, $1500–1749, $1750–1999, $2000–2999 and $3000+. | | | | | | | | | |

| **Model Summary** | | | |
| --- | --- | --- | --- |
|  | -2 Log likelihood | Cox & Snell R Square | Nagelkerke R Square |
|  | 553.377^a^ | .168 | .252 |

| **Hosmer and Lemeshow Test** | | |
| --- | --- | --- |
| Chi-square | df | Sig. |
| 11.313 | 8 | .185 |

### Model 2.2 Outcome: Preferred Option for Choice Set 2 for Non-Concession Card Holders (1=Option A, 0=Option B), N=599

| **Binary Predictor Level of Interest** | **Reference Category** | **B** | **S.E.** | **Wald** | **df** | **Sig.** | **OR** | **95% CI for OR** | |
| --- | --- | --- | --- | --- | --- | --- | --- | --- | --- |
|  |  |  |  |  |  |  |  | **Lower** | **Upper** |
| Reached PBS Safety Net Threshold | Did not reach threshold | .085 | .268 | .101 | 1 | .751 | 1.089 | .644 | 1.842 |
| Lives in major city | Does not live in major city | -.288 | .306 | .887 | 1 | .346 | .750 | .411 | 1.366 |
| Prefers 1–2 months delivery | Prefers 3–6 months | .643 | .210 | 9.390 | 1 | .002 | 1.902 | 1.261 | 2.870 |
| Prefers Pharmacy Pickup | Prefers Delivery | 2.674 | .377 | 50.359 | 1 | <.001 | 14.501 | 6.928 | 30.350 |
| University educated | Not university educated | -.040 | .222 | .032 | 1 | .857 | .961 | .622 | 1.484 |
| Female | Male | -.348 | .212 | 2.697 | 1 | .101 | .706 | .467 | 1.070 |
| Under 65 Years | 65 years and over | -.599 | .275 | 4.766 | 1 | .029 | .549 | .321 | .941 |
| **Continuous Variable** |  |  |  |  |  |  |  |  |  |
| Income |  | -.102 | .041 | 6.302 | 1 | .012 | .903 | .834 | .978 |
| **Constant** |  | .363 | .647 | .315 | 1 | .575 | 1.438 |  |  |
| Notes: For gender, the three responses that were neither male nor female were excluded listwise from this analysis. Weekly income was treated as continuous given the ordinal nature of its collection and spread of results. Discrete continuous categories were: No income, $1–149, $150–299, $300–399, $400–499, $500–649, $650–799, $800–999, $1000–1249, $1250–1499, $1500–1749, $1750–1999, $2000–2999 and $3000+. | | | | | | | | | |

| **Model Summary** | | | |
| --- | --- | --- | --- |
|  | -2 Log likelihood | Cox & Snell R Square | Nagelkerke R Square |
|  | 575.589^a^ | .162 | .240 |

| **Hosmer and Lemeshow Test** | | |
| --- | --- | --- |
| Chi-square | df | Sig. |
| 8.012 | 8 | .432 |

### Model 2.3 Outcome: Preferred Option for Choice Set 3 or Non-Concession Card Holders (1=Option A, 0=Option B), N=599

| **Binary Predictor Level of Interest** | **Reference Category** | **B** | **S.E.** | **Wald** | **df** | **Sig.** | **OR** | **95% CI for OR** | |
| --- | --- | --- | --- | --- | --- | --- | --- | --- | --- |
|  |  |  |  |  |  |  |  | **Lower** | **Upper** |
| Reached PBS Safety Net Threshold | Did not reach threshold | .296 | .255 | 1.345 | 1 | .246 | 1.345 | .815 | 2.218 |
| Lives in major city | Does not live in major city | .236 | .294 | .648 | 1 | .421 | 1.267 | .712 | 2.252 |
| Prefers 1–2 months delivery | Prefers 3–6 months | .612 | .227 | 7.274 | 1 | .007 | 1.844 | 1.182 | 2.876 |
| Prefers Pharmacy Pickup | Prefers Delivery | .759 | .462 | 2.695 | 1 | .101 | 2.135 | .863 | 5.281 |
| University educated | Not university educated | -.093 | .220 | .178 | 1 | .673 | .911 | .592 | 1.402 |
| Female | Male | .203 | .211 | .922 | 1 | .337 | 1.225 | .810 | 1.854 |
| Under 65 Years | 65 years and over | .426 | .259 | 2.713 | 1 | .100 | 1.531 | .922 | 2.541 |
| **Continuous Variable** |  |  |  |  |  |  |  |  |  |
| Income |  | -.041 | .035 | 1.393 | 1 | .238 | .960 | .897 | 1.027 |
| **Constant** |  | -2.733 | .681 | 16.110 | 1 | <.001 | .065 |  |  |
| Notes: For gender, the three responses that were neither male nor female were excluded listwise from this analysis. Weekly income was treated as continuous given the ordinal nature of its collection and spread of results. Discrete continuous categories were: No income, $1–149, $150–299, $300–399, $400–499, $500–649, $650–799, $800–999, $1000–1249, $1250–1499, $1500–1749, $1750–1999, $2000–2999 and $3000+. | | | | | | | | | |

| **Model Summary** | | | |
| --- | --- | --- | --- |
|  | -2 Log likelihood | Cox & Snell R Square | Nagelkerke R Square |
|  | 577.133^a^ | .029 | .046 |

| **Hosmer and Lemeshow Test** | | |
| --- | --- | --- |
| Chi-square | df | Sig. |
| 17.525 | 8 | .025 |

### Model 2.4 Outcome: Preferred Option for Choice Set 4 for Non-Concession Card Holders (1=Option A, 0=Option B), N=598

| **Binary Predictor Level of Interest** | **Reference Category** | **B** | **S.E.** | **Wald** | **df** | **Sig.** | **OR** | **95% CI for OR** | |
| --- | --- | --- | --- | --- | --- | --- | --- | --- | --- |
|  |  |  |  |  |  |  |  | **Lower** | **Upper** |
| Reached PBS Safety Net Threshold | Did not reach threshold | 1.421 | .305 | 21.658 | 1 | <.001 | 4.141 | 2.276 | 7.532 |
| Lives in major city | Does not live in major city | .238 | .424 | .315 | 1 | .575 | 1.268 | .553 | 2.910 |
| Prefers 1–2 months delivery | Prefers 3–6 months | .465 | .316 | 2.162 | 1 | .141 | 1.591 | .857 | 2.956 |
| Prefers Pharmacy Pickup | Prefers Delivery | .115 | .492 | .055 | 1 | .815 | 1.122 | .428 | 2.941 |
| University educated | Not university educated | .010 | .322 | .001 | 1 | .976 | 1.010 | .538 | 1.897 |
| Female | Male | -.208 | .300 | .480 | 1 | .488 | .813 | .452 | 1.461 |
| Under 65 Years | 65 years and over | 1.319 | .492 | 7.195 | 1 | .007 | 3.738 | 1.426 | 9.796 |
| **Continuous Variable** |  |  |  |  |  |  |  |  |  |
| Income |  | -.039 | .051 | .593 | 1 | .441 | .962 | .870 | 1.062 |
| **Constant** |  | -3.943 | .928 | 18.065 | 1 | <.001 | .019 |  |  |
| Notes: For gender, the three responses that were neither male nor female were excluded listwise from this analysis. Weekly income was treated as continuous given the ordinal nature of its collection and spread of results. Discrete continuous categories were: No income, $1–149, $150–299, $300–399, $400–499, $500–649, $650–799, $800–999, $1000–1249, $1250–1499, $1500–1749, $1750–1999, $2000–2999 and $3000+. | | | | | | | | | |

| **Model Summary** | | | |
| --- | --- | --- | --- |
|  | -2 Log likelihood | Cox & Snell R Square | Nagelkerke R Square |
|  | 330.103^a^ | .054 | .120 |

| **Hosmer and Lemeshow Test** | | |
| --- | --- | --- |
| Chi-square | df | Sig. |
| 7.759 | 8 | .457 |

### Model 2.5 Outcome: Preferred Option for Choice Set 5 for Non-Concession Card Holders (1=Option A, 0=Option B), N=597

| **Binary Predictor Level of Interest** | **Reference Category** | **B** | **S.E.** | **Wald** | **df** | **Sig.** | **OR** | **95% CI for OR** | |
| --- | --- | --- | --- | --- | --- | --- | --- | --- | --- |
|  |  |  |  |  |  |  |  | **Lower** | **Upper** |
| Reached PBS Safety Net Threshold | Did not reach threshold | 1.225 | .313 | 15.363 | 1 | <.001 | 3.405 | 1.845 | 6.285 |
| Lives in major city | Does not live in major city | .316 | .443 | .510 | 1 | .475 | 1.372 | .576 | 3.269 |
| Prefers 1–2 months delivery | Prefers 3–6 months | .482 | .323 | 2.228 | 1 | .135 | 1.619 | .860 | 3.050 |
| Prefers Pharmacy Pickup | Prefers Delivery | .302 | .521 | .335 | 1 | .563 | 1.352 | .487 | 3.756 |
| University educated | Not university educated | .345 | .330 | 1.095 | 1 | .295 | 1.412 | .740 | 2.694 |
| Female | Male | -.539 | .309 | 3.039 | 1 | .081 | .583 | .318 | 1.069 |
| Under 65 Years | 65 years and over | 1.133 | .457 | 6.140 | 1 | .013 | 3.106 | 1.267 | 7.614 |
| **Continuous Variable** |  |  |  |  |  |  |  |  |  |
| Income |  | -.062 | .051 | 1.488 | 1 | .222 | .940 | .851 | 1.038 |
| **Constant** |  | -3.807 | .934 | 16.613 | 1 | <.001 | .022 |  |  |
| Notes: For gender, the three responses that were neither male nor female were excluded listwise from this analysis. Weekly income was treated as continuous given the ordinal nature of its collection and spread of results. Discrete continuous categories were: No income, $1–149, $150–299, $300–399, $400–499, $500–649, $650–799, $800–999, $1000–1249, $1250–1499, $1500–1749, $1750–1999, $2000–2999 and $3000+. | | | | | | | | | |

| **Model Summary** | | | |
| --- | --- | --- | --- |
|  | -2 Log likelihood | Cox & Snell R Square | Nagelkerke R Square |
|  | 324.709^a^ | .048 | .108 |

| **Hosmer and Lemeshow Test** | | |
| --- | --- | --- |
| Chi-square | df | Sig. |
| 14.327 | 8 | .074 |

### Model 2.6 Outcome: Preferred Option for Choice Set 6 for Non-Concession Card Holders (1=Option A, 0=Option B), N=597

| **Binary Predictor Level of Interest** | **Reference Category** | **B** | **S.E.** | **Wald** | **df** | **Sig.** | **OR** | **95% CI for OR** | |
| --- | --- | --- | --- | --- | --- | --- | --- | --- | --- |
|  |  |  |  |  |  |  |  | **Lower** | **Upper** |
| Reached PBS Safety Net Threshold | Did not reach threshold | .952 | .324 | 8.635 | 1 | .003 | 2.590 | 1.373 | 4.886 |
| Lives in major city | Does not live in major city | .627 | .502 | 1.557 | 1 | .212 | 1.871 | .699 | 5.006 |
| Prefers 1–2 months delivery | Prefers 3–6 months | .586 | .329 | 3.179 | 1 | .075 | 1.797 | .943 | 3.423 |
| Prefers Pharmacy Pickup | Prefers Delivery | .350 | .520 | .452 | 1 | .502 | 1.418 | .512 | 3.930 |
| University educated | Not university educated | .505 | .338 | 2.230 | 1 | .135 | 1.658 | .854 | 3.218 |
| Female | Male | -.515 | .310 | 2.760 | 1 | .097 | .597 | .325 | 1.097 |
| Under 65 Years | 65 years and over | 1.916 | .612 | 9.806 | 1 | .002 | 6.792 | 2.048 | 22.528 |
| **Continuous Variable** |  |  |  |  |  |  |  |  |  |
| Income |  | -.063 | .052 | 1.500 | 1 | .221 | .938 | .848 | 1.039 |
| **Constant** |  | -4.920 | 1.060 | 21.561 | 1 | <.001 | .007 |  |  |
| Notes: For gender, the three responses that were neither male nor female were excluded listwise from this analysis. Weekly income was treated as continuous given the ordinal nature of its collection and spread of results. Discrete continuous categories were: No income, $1–149, $150–299, $300–399, $400–499, $500–649, $650–799, $800–999, $1000–1249, $1250–1499, $1500–1749, $1750–1999, $2000–2999 and $3000+. | | | | | | | | | |

| **Model Summary** | | | |
| --- | --- | --- | --- |
|  | -2 Log likelihood | Cox & Snell R Square | Nagelkerke R Square |
|  | 314.893^a^ | .056 | .127 |

| **Hosmer and Lemeshow Test** | | |
| --- | --- | --- |
| Chi-square | df | Sig. |
| 5.635 | 8 | .688 |
